# Diabetes

**Published:** 1905-10-14

**Authors:** 


### DIABETES.

**Definition.**—The obscurity in which our present knowledge of this condition is involved renders an exact definition almost impossible. As Bosanquet[1] points out in his exhaustive survey of the subject, the[1] name diabetes was first indiscriminately applied to all conditions characterised by polyuria; later the form in which sugar was found in the urine was separated off as a morbid entity, and at present we must restrict the name diabetes mellitus still further, and leaving on one side the glycosuria accompanying cerebral injuries, certain nervous diseases, exophthalmic goitre, etc., apply it only to cases when the sugar is passed in considerable amount and for long periods. Unsatisfactory as such a definition must be, it is the best which our scanty knowledge at present allows us to make. Bosanquet lays great stress upon the fact that what is known as "alimentary" diabetes may pass into the more severe form called "composite" by Pavy. In the latter form sugar appears in the urine irrespective of diet, and in fact continues to appear during starvation, being derived from the destruction of the body tissues. This fact, he considers, gives the real basis for the definition of a true diabetes—namely, a disease in which sugar appears in the urine as a result of destructive changes (the setting free of sugar) in the tissues. In the early stages, or in slighter cases, the cells continue to be able to assimilate a certain amount of sugar, whether supplied in the food or from an internal katabolic source. If, however, this amount is exceeded, glycosuria results. Hence in these cases limiting the amount of carbohydrate food stops the glycosuria. As the case progresses, or in very severe cases from the first, the amount of sugar formed internally is so great that it cannot be dealt with by the body; there is always an unused residue, even when no carbohydrate is supplied in the food, and hence there is always glycosuria. This view, he considers, has the advantage of explaining by one and the same morbid process (namely, increased internal sugar production) both the alimentary and composite forms of diabetes, Pavy[2] holds a different view. He sees no objection to supposing that two pathological processes underlie the two forms of diabetes. In "alimentary diabetes" he thinks there is merely a failure to absorb the sugar taken as food, which thus passes in solution into the blood and filters out through the kidneys. On the other hand, "composite" diabetes, as the name he has given it suggests, consists not only of a failure to absorb sugar, but also of a breaking-down process, by which extra sugar is set free in the blood from the tissues.

**Causation.**—If the essential nature of diabetes is still uncertain, its ætiology is no less obscure. Bosanquet enumerates various conditions such as excess in alcohol or tobacco which may play their part in some cases, lays some stress upon heredity as having a distinct influence. He also quotes Lorand in support of a racial influence, Jews being according to that author much more liable than western nations. These two factors, and the influence of certain acute infections are indeed now generally recognised as causative factors. The evidence that true diabetes is in itself an infective process is not, however, as yet very convincing. Injuries, especially those involving the nervous system, and mental disease are not regarded by Bosanquet as clearly established ætiological factors, though in some cases they appear to have given rise to a true diabetes. The comparative rarity of diabetes and the frequency of the accidents and mental conditions which have been thought to give rise to it are facts which show that there must be some further element in its causation at present unrecognised. Two further points remain to be noticed, firstly the production of diabetes by phlorizin, and secondly the rôle of the pancreas in this disease. Phlorizin, a glucoside derived from the cherry and other trees, produces, as Bosanquet shows, a typical diabetes. Thirst, polyuria, glycosuria, and ultimately coma, develop; there are marked fatty changes in the liver, and the acetone bodies are excreted in large amounts in the urine. The only point in which this differs from true diabetes is that there is little if any excess of sugar in the blood. Some authorities say that the sugar content of the blood is actually below normal, but Pavy, who has made extensive experiments with phlorizin, says that these statements are due to faulty experimental methods, and that there is always a slight excess of sugar in the blood. This curious fact is explained by some as being due to the action of phlorizin in damaging the renal epithelium, thereby allowing sugar to pass through it, and the glycosuria of Bright's disease has been adduced as due to a similar condition. This, however, is inadequate in view of the blood condition. Pavy ascribes the process as due to a direct action on the renal epithelial cells, whereby they split off from the proteids of the blood their sugar component, which then passes into the urine. The relationship of pancreatic disease to diabetes is very fully dealt with by Bosanquet. His main conclusions are that it has been proved that some connection exists between these conditions in many cases, and is probably present in all; he thinks that the evidence that Langerhans' Islands are the portion of the pancreas intimately concerned is increasing, and will ultimately be firmly established. As to how the pancreas acts in the matter, he states that there is no evidence that this gland furnishes an internal secretion necessary to the assimilation of sugar, but there is some evidence that it may furnish a substance which neutralises a poison formed elsewhere, the action of which is to cause a hyperglycæmia. Pavy's experiments are based on a different view. He considers that the pancreas furnishes an "activator," or substance which enables the blood proteids to combine with the carbohydrate molecules. In support of this theory he injected various sugars intravenously into rabbits, and by a special method which he has devised, estimated the amount of carbohydrate which could be split off from the blood proteids. He then made another series of experiments, in which, in addition to the sugars, boiled pancreatic extract was injected. The blood from these rabbits when tested was found to contain a much higher percentage of carbohydrate molecules. As an analogous reaction, Pavy quotes the experiments of Holden and Young. Enzymes are regarded as dual bodies, containing two active substances, one insoluble in alcohol, destroyed by heat, and non-diffusable, the other the co-ferment or activator, which is usually soluble in alcohol, diffusable, and not destroyed by boiling. By adding an equal volume of boiled yeast juice to the raw expressed yeast juice the experimenters obtained a doubly strong ferment action. Pavy's boiled pancreatic extract contained, he thinks, a similar activator or co-ferment.

[1] Lancet, i., 1905, pp. 903, 977, 1053. [2] Ibid., i., 1905, p. 1704; ii., 1905, pp. 4, 342.

